# The mediating role of physical activity and sedentary behavior in the association between working from home and musculoskeletal pain during the COVID-19 pandemic

**DOI:** 10.3389/fpubh.2022.1072030

**Published:** 2022-12-02

**Authors:** Bette Loef, Sandra H. van Oostrom, Esmee Bosma, Lifelines Corona Research Initiative, H. Marike Boezen, Karin I. Proper

**Affiliations:** ^1^Center for Nutrition, Prevention and Health Services, National Institute for Public Health and the Environment, Bilthoven, Netherlands; ^2^Department of Public and Occupational Health, Amsterdam University Medical Center, Vrije Universiteit Amsterdam, Amsterdam Public Health Research Institute, Amsterdam, Netherlands

**Keywords:** COVID-19, home workers, hybrid workers, longitudinal study, mediation, musculoskeletal pain, physical activity, sedentary behavior

## Abstract

**Introduction:**

Working from home during the COVID-19 pandemic has been associated both with physical inactivity and musculoskeletal pain. However, it has not been examined whether physical activity and sedentary behavior are underlying mechanisms in the association between working from home and musculoskeletal pain. Therefore, we examined their mediating role in this association.

**Methods:**

Data were used from 24 questionnaire rounds of the Lifelines COVID-19 cohort (March 2020–January 2022). Longitudinal information on work situation (location, home, hybrid), physical activity, sedentary behavior, and musculoskeletal pain was collected among 28,586 workers. Analysis of physical activity/sedentary behavior as mediators of the association between working from home and musculoskeletal pain was performed using multilevel structural equation modeling.

**Results:**

Home workers more often had pain in the upper back [odds ratio (OR) = 1.17, 95%-confidence interval (CI) = 1.02–1.34] and arm, neck, and/or shoulder (ANS) (OR = 1.32, 95%-CI = 1.19–1.47) than location workers. Furthermore, home workers were more often sedentary for >9 h per work day than location workers (OR = 2.82, 95%-CI = 2.56–3.09), and being more sedentary was associated with musculoskeletal pain (upper back: OR = 1.17, 95%-CI = 1.06–1.30; ANS: OR = 1.25, 95%-CI = 1.16–1.34). Corresponding indirect effects were OR = 1.18 (95%-CI = 1.04–1.33) and OR = 1.26 (95%-CI = 1.12–1.35). No indirect effect was found for physical activity. Similar indirect effects were observed for hybrid workers.

**Conclusion:**

Home and hybrid workers were more likely to have pain in the upper musculoskeletal system during the COVID-19 pandemic than location workers, which was partly mediated by increased sedentary behavior, but not by reduced physical activity. Measures to reduce sedentary time in home workers may contribute to preventing musculoskeletal pain.

## Introduction

During the COVID-19 pandemic, countries around the world implemented measures to contain the spread of the SARS-CoV-2 virus (1). As part of these containment measures, the Dutch government as well as many other governments, asked workers to work from home as much as possible. Although the work-from-home measure contributed to reducing SARS-CoV-2 infections, the large changes in workers' daily routine and work environment may also have had a negative impact on the lifestyle and health of these workers (2–4).

Regarding home workers' lifestyle, being confined to the house may have resulted in less physically activity and more sedentary behavior during the COVID-19 pandemic (5–10). Earlier studies during the first year of the pandemic have observed an association of working from home with reduced physical activity and increased sedentary behavior (5–10). In addition, in our recent longitudinal study among 33,325 Dutch workers, we found home workers during the first year of the COVID-19 pandemic to be less likely to be physically active and more likely to be sedentary than location workers (11).

Even before the COVID-19 pandemic, the physical health of workers has been suggested to be negatively impacted by working from home (12). During the COVID-19 pandemic, some studies (13–17), but not all (18, 19), found a positive association between working from home and musculoskeletal pain. We previously found Dutch home workers to be more likely to report musculoskeletal pain than location workers during the first year of the COVID-19 pandemic (20). Home workers might experience increased musculoskeletal pain as a result of multiple factors, such as an unfavorable working environment at home with continuous repetitive movements and increased psychosocial risks (12, 21). Furthermore, reduced physical activity and increased sedentary behaviors can potentially explain part of the association between working from home and musculoskeletal pain (4) by causing physiological changes such as altering muscle activity and strength, increasing strain on the musculoskeletal system, and augmenting pain sensitivity (22–26).

However, research on the mediating role of physical activity and sedentary behavior in the association between working from home and musculoskeletal pain during the COVID-19 pandemic is currently lacking. This may be due to the novelty of the global pandemic and the fact that longitudinal data is required to study this role. Nevertheless, studying this mediating role is important, because it can contribute to the understanding of how working from home is linked to musculoskeletal pain, and knowledge about underlying mechanisms may provide starting points on preventing these complaints in home workers. Prevention of health problems in home workers is especially valuable since even after the COVID-19 containment measures have been lifted, working from home arrangements have remained partly in place and expectations are that these arrangements will continue to exist in the future (21, 27).

Therefore, the aim of the current study was to examine the mediating role of physical activity and sedentary behavior in the association between working from home and musculoskeletal pain during almost 2 years of the COVID-19 pandemic.

## Methods

### Study design and population

In the current longitudinal study, data were used from the Lifelines COVID-19 cohort. The Lifelines COVID-19 cohort investigates factors relevant to the impact of the COVID-19 pandemic among a population-based cohort living in the Northern Netherlands (28). Since the beginning of the pandemic in March 2020, participants have regularly completed digital questionnaires with questions on health, lifestyle, work, and attitude toward the pandemic. Questionnaires were sent out on a (bi)weekly basis until July 2020, and on a monthly basis from July 2020 onwards. Response rates varied between 28 and 49%.

Participants of the Lifelines COVID-19 cohort were recruited from the Lifelines population cohort. The Lifelines population cohort is a multi-disciplinary prospective population-based cohort study examining in a unique three-generation design the health and health-related behaviors of 167,729 persons living in the north of the Netherlands (29, 30). It employs a broad range of investigative procedures in assessing the biomedical, socio-demographic, behavioral, physical and psychological factors which contribute to the health and disease of the general population, with a special focus on multi-morbidity and complex genetics. Inhabitants from the north of the Netherlands were recruited to participate in the Lifelines population cohort through their general practitioner, their family members, or self-registration. Approval of the study was obtained from the Medical Ethics Committee of the University Medical Center Groningen, The Netherlands (number 2007/152). Written informed consent was obtained from all participants. All adult participants from the Lifelines population cohort with known email addresses were asked to participate in the Lifelines COVID-19 cohort (*n* = 140,145) (28).

Inclusion criteria for the current study were being an active worker for the majority of the study (see section Work situation), being aged between 18 and 67 years at baseline, and having information available on work situation, physical activity, and musculoskeletal pain. In the current study, data were used from 24 questionnaire rounds of the Lifelines COVID-19 cohort conducted between March 2020 and January 2022 (Supplementary Table S1).

### Work situation

In the questionnaire rounds, participants were asked what they currently did in their daily life (student; work; on disability; unemployed; retired; maternity leave; other). Next, working participants were asked to report their current work situation from one or more of the following responses: I work from home; I am laid off but am still being paid; I am laid off and am no longer being paid; I continue to work at the usual location (e.g., office, factory, construction site); I continue to work at multiple sites for my job; I am forced to take sick leave or vacation time; other. Based on this question, a time-dependent variable for work situation was constructed. If participants indicated to work at the usual location and/or at multiple sites for their job they were considered location workers for that particular round, if they indicated to work from home they were considered home workers, and if they indicated to work on location as well as from home they were considered hybrid workers. For hybrid workers, the distribution of work time between remote work and location work was unknown. As we aimed to include participants who were active workers for the majority of the study, only workers who worked >75% of the rounds in which they participated were included. In addition, of the rounds in which they worked, workers needed to work >75% of the time on location and/or from home to be included. These criteria were formulated to ensure that in general only active workers were included in the study in order to provide results that were applicable to home workers, hybrid workers, and location workers.

### Physical activity and sedentary behavior

To assess physical activity, participants were asked “how many minutes of (moderately) intense activity did you do (e.g., walking, biking or running)” in the last 7 days (rounds 1–6) or 14 days (round 7 onwards). Answer categories were < 50; 50–100; 100–150; 150–180; >180 min in the last 7 days or < 100; 100–200; 200–300; 300–360; >360 min in the last 14 days. Subsequently, answers were dichotomized into being physically active for ≥150 vs. < 150 min per week to correspond with the global physical activity recommendation (31).

Sedentary behavior was assessed by asking participants how much time they on average spent sitting per work day in the last 7 days (round 6) or 14 days (round 11 onwards). Answer categories were < 1; 1; 2; 3; 4; 5; 6; 7; 8; 9; 10; 11; 12; >12 h (until round 17) or < 1; 1–3; 4–6; 7–9; 10–12; >12 h (from round 19 onwards). Subsequently, answers were dichotomized into sitting >9 vs. ≤ 9 h per work day based on the average sitting time of the Dutch population in 2021 (32). Sedentary behavior is different between work days and weekend days among Dutch workers (32). In the current study, we were specifically interested in sedentary behavior on work days, since this is likely to be the most impacted by working from home.

The questions on physical activity and sedentary behavior in the Lifelines COVID-19 cohort were based on the Dutch Physical Activity Guidelines 2017 and the International Physical Activity Questionnaire Short Form (IPAQ-SF) (33, 34).

### Musculoskeletal pain

Musculoskeletal pain was assessed by asking participants to what extent they had pain in the lower back in the last 7 days (rounds 1–6) or 14 days (round 7 onwards). From round 8 onwards, participants were also asked about pain in the upper back in the last 14 days in one question and about pain in the arm, neck, and/or shoulder in the last 14 days in another question. Answer categories of the questions on pain were not at all; a little bit; somewhat; quite a lot; very much. Subsequently, answers were dichotomized into no pain vs. pain (a little bit to very much). The questions on pain were based on the Symptom CheckList-90 Somatization scale (35).

### Covariates

The following covariates were included: age, sex, education level, country of birth, household composition, occupation and occupational class (both based on the International Standard Classification of Occupations), employment contract, general health, physical activity before the pandemic, and sedentary behavior before the pandemic. Age, sex, education level, country of birth, occupation, and occupational class were derived from the Lifelines population cohort. Household composition and employment contract were measured in round 1–23 of the Lifelines COVID-19 cohort, and general health in round 1–2. Physical activity and sedentary behavior before the pandemic was assessed by asking participants in round 1–2 how many minutes of (moderately) intense activity they performed each week before the COVID-19 pandemic and how much time they spent sitting on average per work day before the COVID-19 pandemic. More information on the included covariates can be found elsewhere (11).

### Statistical analysis

The independent-samples *t*-test and the chi-square test were used to compare the characteristics of the study population by work situation. To this end, a fixed variable for work situation based on the work situation in the entire follow-up from March 2020 to January 2022 was constructed. In this variable, location workers worked on location and did not work from home during the entire follow-up, home workers worked from home and did not work on location during the entire follow-up, and hybrid workers worked both on location and from home during the entire follow-up (but this did not necessarily had to be at the same time/questionnaire round, which is the case for the time-dependent work situation variable).

Six longitudinal mediation models were constructed where work situation and the two potential mediators (physical activity/sedentary behavior) measured at round *t* were studied in relation to the three pain outcomes (pain in lower back; upper back; arm, neck, and/or shoulder) measured at round *t* + 1 separately. Because current work situation is expected to have an immediate impact on physical activity and sedentary behavior and because it is generally unlikely that physical activity and sedentary behavior impact work situation, the determinant and mediator were assessed at the same round. Since it may be expected that some time is needed for physical activity and sedentary behavior to have an impact on pain outcomes and in order to reduce the possibility of reversed causality, the outcome was assessed at every subsequent round after the round in which the determinant and mediator were assessed. Supplementary Table S1 shows an overview of the used information on determinant, mediators, and outcomes from each questionnaire round.

**Figure 1:**
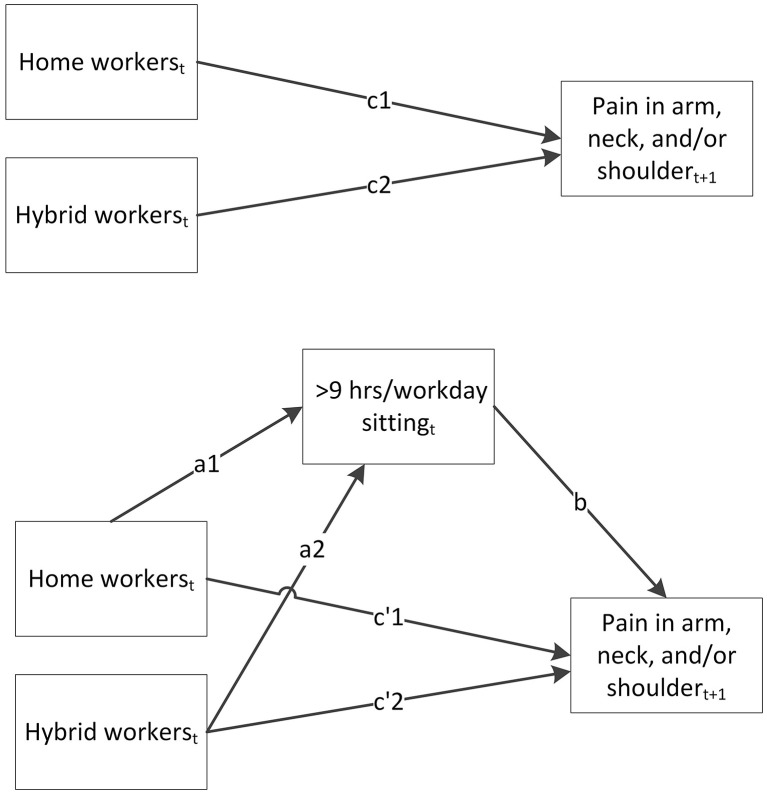
Longitudinal mediation model of the total effect (c1 and c2) of work situation measured in round *t* on pain in the arm, neck, and/or shoulder measured in round *t* + 1, the indirect effect of sedentary behavior measured in round t (a1*b and a2*b), and the direct effect (c'1 and c'2) of work situation on pain in the arm, neck, and/or shoulder.

Structural equation modeling (SEM) was used to estimate the different paths of the mediation models. In Figure 1, these different paths are shown for one of the longitudinal mediation models. The upper part of this figure shows the model of the total effect (c1 and c2) of work situation on pain in the arm, neck, and/or shoulder measured in the next round. The lower part shows the indirect effect of sedentary behavior (a1^*^b and a2^*^b), and the direct effect (c'1 and c'2) of work situation on pain in the arm, neck, and/or shoulder that is independent of sedentary behavior. To calculate the values of these paths, multilevel SEM was conducted with logistic regression adjusting for the dependency of observations within an individual over time (36). Age, sex, education level, country of birth, household composition, occupation, occupational class, employment contract, general health, physical activity before the pandemic, and sedentary behavior before the pandemic were included as potential confounders in the analyses. Indirect effects were calculated by taking the product of the a-path and b-path (37). For the indirect effects, a 95% bootstrap confidence interval was calculated using 500 bootstrap resamples.

Descriptive analyses were conducted using IBM SPSS Statistics, version 25 (IBM Corporation, Armonk, NY, USA). SEM analyses were performed using Stata/SE, version 13 (StataCorp LLC, College Station, TX, USA). A two-sided *p*-value of < 0.05 was considered statistically significant.

## Results

### Study population

In total, 76,424 adults participated in the Lifelines COVID-19 cohort (Supplementary Figure S1). Of those, 43,281 participants were aged 18–67 years, employed >75% of their follow-up time, and working >75% of their time employed on location and/or from home. After excluding participants with missing data on physical activity, pain in the lower back (in a round subsequent to a round in which work situation and physical activity was measured), and covariates, 28,586 participants were included in the current study. Because pain in the upper back and in the arm, neck, and/or shoulder was assessed in fewer rounds than pain in the lower back, and because sedentary behavior was assessed in fewer rounds than physical activity, fewer participants were available for the analyses with these outcomes and mediator. Of the total of 28,586 participants, 20,354 participants were included for the mediation models with physical activity as mediator and pain in the upper back/pain in the arm, neck, and/or shoulder as outcomes. Furthermore, 16,780 and 14,333 participants were included for the mediation models with sedentary behavior as mediator and pain in the lower back and pain in the upper back/pain in the arm, neck, and/or shoulder, respectively, as outcomes (Supplementary Figure S1).

An overview of the characteristics of the study population can be found in Supplementary Table S2. Table 1, which presents a selection of these characteristics, shows that home and hybrid workers were younger than location workers (49.3 and 49.1 years vs. 50.9 years). Furthermore, home and hybrid workers more often had a high education level (65.8 and 64.7% vs. 23.5%) and a high-skilled white-collar occupation (73.2 and 72.4% vs. 42.2%) than location workers. Compared to location workers, home and hybrid workers reported somewhat less often to have a good or excellent health (95.3 and 95.9% vs. 96.5%). Before the pandemic, home workers were more often physically active for ≥150 min per week (44.6 vs. 41.9%) than locations workers, but they were also more often sitting >9 h per work day (43.3 vs. 12.1%) than location workers. Since Table 1 and Supplementary Table S2 present descriptive information, these differences in pre-pandemic physical activity and sedentary behavior are likely to be related to differences in demographic and work-related factors between home workers and location workers.

**Table 1:** Characteristics of the study population stratified for location workers, home workers, and hybrid workers (who worked both on location and from home) during the COVID-19 pandemic from March 2020 to January 2022 (*n* = 28,586).

	**Location workers** **(*****n*** = **13,489)**		**Home workers** **(*****n*** = **5,971)**		**Hybrid workers** **(*****n*** = **9,126)**	
	**Mean/%**	**SD/*n***	**Mean/%**	**SD/*n***	**Mean/%**	**SD/*n***
Age (in years)	50.9	8.6	49.3^*^	9.1	49.1 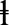	9.3
Sex (% female)	61.3	8,264	56.6^*^	3,377	60.3	5,503
**Education level (%)**						
Low	20.8	2,807	5.3^*^	317	4.9 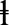	445
Middle	55.7	7,511	28.9^*^	1,724	30.4 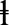	2,778
High	23.5	3,171	65.8^*^	3,930	64.7 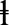	5,903
**Occupation (%)**						
High-skilled white-collar	42.2	5,693	73.2^*^	4,373	72.4 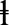	6,605
Low-skilled white-collar	34.2	4,607	23.2^*^	1,388	22.3 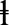	2,039
High-skilled blue-collar	11.2	1,515	2.1^*^	128	3.5 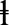	323
Low-skilled blue-collar	12.4	1,674	1.4^*^	82	1.7 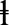	159
General health (% excellent/(very) good)	96.5	13,019	95.3^*^	5,693	95.9 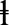	8,748
≥150 min of physical activity per week before COVID-19 pandemic (% yes)	41.9	5,651	44.6^*^	2,662	42.1	3,838
Sitting >9 h per work day before COVID-19 pandemic (% yes)^a^	12.1	913	43.3^*^	1,274	30.4 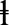	1,902

* Statistically significant difference (*p* < 0.05) between home workers and location workers tested with independent-samples *t*-test and chi-square test.

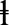
Statistically significant difference (*p* < 0.05) between hybrid workers and location workers tested with independent-samples *t*-test and chi-square test.

a Among 7,573 locations workers, 2,943 home workers, and 6,264 hybrid workers.

The percentage of the study population who worked exclusively on location ranged between 50% during the first questionnaire round (March–April 2020) and 71% in October–November 2021, while the percentage of exclusive home workers ranged between 44% in March–April 2020 and 16% in October–November 2021 (Supplementary Figure S2). A minority of the study population were workers who worked both on location and from home in a particular round, with percentages ranging between 3% in April–May 2020 and 13% in October–November 2021.

The percentage of workers with pain in the lower back, in the upper back, and in the arm, neck, and/or shoulder were, respectively, 35, 13, and 33% on average over time. These percentages were similar for each work situation.

### Working from home, physical activity, and musculoskeletal pain

Table 2 shows the results of the longitudinal mediation models for the association between work situation and pain with physical activity as potential mediator. Home workers (OR = 1.02, 95%-CI = 0.97–1.08) and hybrid workers (OR = 1.05, 95%-CI = 0.97–1.13) did not have higher odds of having pain in the lower back than location workers. However, they did have higher odds of having pain in the upper back (home workers: OR = 1.12, 95%-CI = 1.01–1.25; hybrid workers: OR = 1.16, 95%-CI = 1.02–1.32) and pain in the arm, neck, and/or shoulder (home workers: OR = 1.19, 95%-CI = 1.10–1.29; hybrid workers: OR = 1.21, 95%-CI = 1.10–1.34) than location workers. After taking physical activity into account as potential mediator, the same associations were observed. Home workers (OR = 0.87, 95%-CI = 0.83–0.91), but not hybrid workers (OR = 1.04, 95%-CI = 0.98–1.10), were less likely to be physically active for ≥150 min per week than location workers in the total study population. However, being physically active for ≥150 min per week was not associated with any type of pain (lower back: OR = 0.97, 95%-CI = 0.94–1.01; upper back: OR = 1.04, 95%-CI = 0.98–1.11; arm, neck, and/or shoulder: OR = 1.00, 95%-CI = 0.95–1.04). Therefore, no indirect effect of physical activity on the association between work situation and pain was observed (OR = 1.00 for all types of pain).

**Table 2:** Odds ratios of the total effect and direct effect of work situation (WS) on pain, the association between work situation and physical activity (PA), the association between physical activity and pain, and the indirect effect of physical activity.

	**Pain in the lower back**						**Pain in the upper back**						**Pain in the arm, neck, and/or shoulder**					
	***N*** = **28,586, observations** = **211,141**						***N*** = **20,354, observations** = **107,161**						***N*** = **20,354, observations** = **107,161**					
	**Home workers**			**Hybrid workers**			**Home workers**			**Hybrid workers**			**Home workers**			**Hybrid workers**		
	**OR**	**95%-CI**		**OR**	**95%-CI**		**OR**	**95%-CI**		**OR**	**95%-CI**		**OR**	**95%-CI**		**OR**	**95%-CI**	
c-path (total effect)	1.02	0.97	1.08	1.05	0.97	1.13	1.12^*^	1.01	1.25	1.16^*^	1.02	1.32	1.19^*^	1.10	1.29	1.21^*^	1.10	1.34
c'-path (direct effect)	1.02	0.97	1.08	1.05	0.97	1.13	1.12^*^	1.01	1.25	1.16^*^	1.02	1.32	1.19^*^	1.10	1.29	1.21^*^	1.10	1.34
a-path (WS -> PA)	0.87^*^	0.83	0.91	1.04	0.98	1.10	1.03	0.97	1.10	1.09^*^	1.01	1.18	1.03	0.97	1.10	1.09^*^	1.01	1.18
b-path (PA -> pain)	0.97	0.94	1.01	0.97	0.94	1.01	1.04	0.98	1.11	1.04	0.98	1.11	1.00	0.95	1.04	1.00	0.95	1.04
Indirect effect (a^*^b)	1.00	1.00	1.01	1.00	1.00	1.00	1.00	0.99	1.01	1.00	1.00	1.01	1.00	1.00	1.00	1.00	1.00	1.00

CI, confidence interval; OR, odds ratio; PA, physical activity; WS, work situation.

Odds ratios are adjusted for age, sex, education level, country of birth, household composition, occupation, occupational class, employment contract, general health, and physical activity before the pandemic.

* *p* < 0.05.

### Working from home, sedentary behavior, and musculoskeletal pain

**Figure 2:**
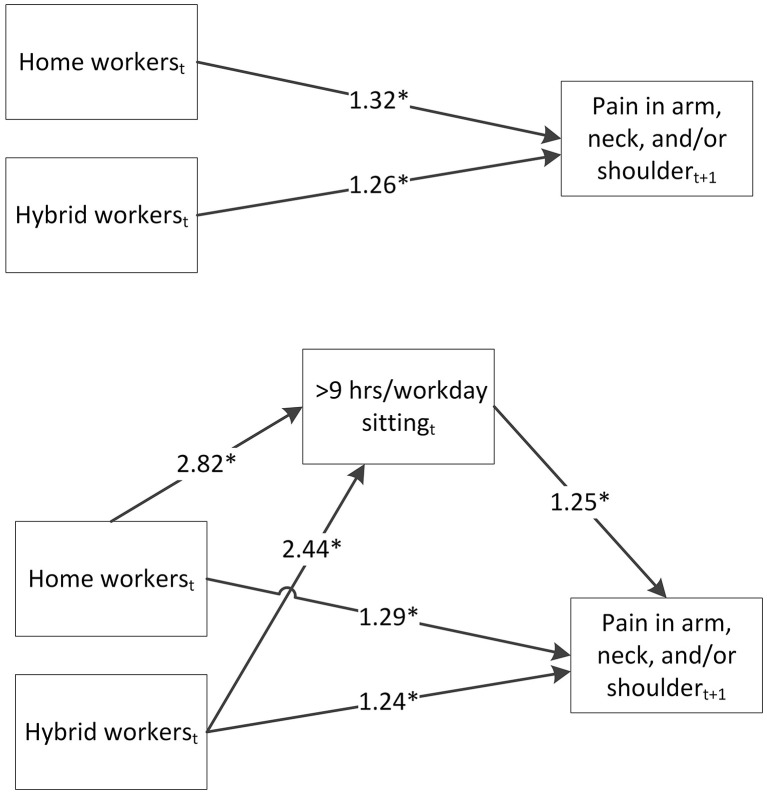
Longitudinal mediation model showing the odds ratios of the total effect of work situation measured in round *t* on pain in the arm, neck, and/or shoulder measured in round *t* + 1, the indirect effect of sedentary behavior measured in round *t*, and the direct effect of work situation on pain in the arm, neck, and/or shoulder (*n* = 14,333). Odds ratios are adjusted for age, sex, education level, country of birth, household composition, occupation, occupational class, employment contract, general health, and sedentary behavior before the pandemic. *p < 0.05.

Table 3 shows the results of the longitudinal mediation models for the association between work situation and pain with sedentary behavior as potential mediator. Similar as in the models with physical activity, home workers and hybrid workers were more likely to have pain in the upper back and pain in the arm, neck, and/or shoulder than location workers. Including sedentary behavior as mediator in the models somewhat reduced the effect estimates between work situation and all types of pain. Home workers (OR = 2.82, 95%-CI = 2.56–3.09; *n* = 14,333) and hybrid workers (OR = 2.44, 95%-CI = 2.18–2.74; *n* = 14,333) were more likely to be sedentary for >9 h per work day than locations workers. Furthermore, workers who were sedentary for >9 h per work day were more likely to have pain in the lower back (OR = 1.12, 95%-CI = 1.05–1.20), in the upper back (OR = 1.17, 95%-CI = 1.06–1.30) and pain in the arm, neck, and/or shoulder (OR = 1.25, 95%-CI = 1.16–1.34) than workers who were sedentary for ≤ 9 h/work day. As a result, statistically significant indirect effects of sedentary behavior on the association between work situation and all types of pain were observed (ORs ranging from 1.09 to 1.26). For example, home workers had a 1.26 higher odds (95%-CI = 1.12–1.35) of having pain in the arm, neck, and/or shoulder than location workers *via* being more often sedentary for >9 h per work day. Figure 2 shows the longitudinal mediation model with sedentary behavior as mediator and pain in the arm, neck, and/or shoulder as outcome with the odds ratios for the different paths.

**Table 3:** Odds ratios of the total effect and direct effect of work situation (WS) on pain, the association between work situation and sedentary behavior (SB), the association between sedentary behavior and pain, and the indirect effect of sedentary behavior.

	**Pain in the lower back**						**Pain in the upper back**						**Pain in the arm, neck, and/or shoulder**					
	***N*** = **16,780, observations** = **80,056**						***N*** = **14,333, observations** = **65,927**						***N*** = **14,333, observations** = **65,927**					
	**Home workers**			**Hybrid workers**			**Home workers**			**Hybrid workers**			**Home workers**			**Hybrid workers**		
	**OR**	**95%-CI**		**OR**	**95%-CI**		**OR**	**95%-CI**		**OR**	**95%-CI**		**OR**	**95%-CI**		**OR**	**95%-CI**	
c-path (total effect)	1.04	0.96	1.14	1.07	0.96	1.19	1.17^*^	1.02	1.34	1.22^*^	1.04	1.45	1.32^*^	1.19	1.47	1.26^*^	1.12	1.43
c'-path (direct effect)	1.03	0.95	1.13	1.06	0.95	1.18	1.15	1.00	1.32	1.20^*^	1.02	1.42	1.29^*^	1.16	1.43	1.24^*^	1.09	1.40
a-path (WS -> SB)	2.44^*^	2.26	2.64	2.16^*^	1.95	2.38	2.82^*^	2.56	3.09	2.44^*^	2.18	2.74	2.82^*^	2.56	3.09	2.44^*^	2.18	2.74
b-path (SB -> pain)	1.12^*^	1.05	1.20	1.12^*^	1.05	1.20	1.17^*^	1.06	1.30	1.17^*^	1.06	1.30	1.25^*^	1.16	1.34	1.25^*^	1.16	1.34
Indirect effect (a^*^b)	1.11^*^	1.02	1.17	1.09^*^	1.02	1.14	1.18^*^	1.04	1.32	1.15^*^	1.03	1.27	1.26^*^	1.12	1.35	1.22^*^	1.10	1.30

CI, confidence interval; OR, odds ratio; SB, sedentary behavior; WS, work situation.

Odds ratios are adjusted for age, sex, education level, country of birth, household composition, occupation, occupational class, employment contract, general health, and sedentary behavior before the pandemic.

* *p* < 0.05.

## Discussion

In the current study among a large population of Dutch workers, we examined the mediating role of physical activity and sedentary behavior in the association between working from home and musculoskeletal pain during almost 2 years of the COVID-19 pandemic. Home workers and hybrid workers were more likely to experience pain in the upper back and in the arm, neck, and/or shoulder than location workers. This association was mediated by increased sedentary behavior among home and hybrid workers, but not by reduced physical activity.

Sedentary behavior was found to explain part of the association between working from home and musculoskeletal pain during the COVID-19 pandemic. Home and hybrid workers were more likely to be sedentary for >9 h per work day than locations workers, and being more sedentary was statistically significantly associated with having musculoskeletal pain. Similarly, earlier studies during the COVID-19 pandemic have observed working from home to be associated with increased sedentary behavior (6–9, 11). Furthermore, three recent systematic reviews reported sedentary behavior (38, 39) and screen time (40) to be associated with musculoskeletal pain, possibly due to increased static load on the musculoskeletal system (39, 41). The observed indirect effects of sedentary behavior in the current study cannot be compared with earlier work, because no mediation analysis has been performed up until now. Earlier studies conducted during the COVID-19 pandemic often lacked information on either working from home, sedentary behavior, or musculoskeletal pain, lacked a sufficiently large sample size, and/or lacked a longitudinal design, while all of these criteria are needed in order to perform a mediation analysis. Nevertheless, our results together with the observed association between working from home and sedentary behavior (a-paths) and between sedentary behavior and musculoskeletal pain (b-paths) in other studies suggest that sedentary behavior may play an important mechanistic role in the association between working from home and musculoskeletal pain.

Physical activity, defined as being physically active for ≥150 min per week, was not found to be a mediator in the association between working from home and musculoskeletal pain. This can be explained by the finding that physical activity was not associated with musculoskeletal pain. Previous systematic reviews have reported conflicting results regarding this association, with two reporting evidence supporting an association between some types of physical activity and musculoskeletal pain (42, 43), while another one does not (44). With respect to the association between working from home and physical activity, we found home workers to be less likely to be physically active for ≥150 min per week than location workers in the total study population, but this association disappeared in the smaller subsamples of the population. In earlier work during the COVID-19 pandemic, most (5–7, 11), but not all (9), studies observed an association between working from home and reduced physical activity. Possibly, other measures of physical activity than the relatively crude measure of adhering to the global physical activity recommendation that was used in the current study, may still be valuable in explaining the association between working from home and musculoskeletal pain. This is supported by the finding that in particular vigorous-intensity activity has the potential to reduce musculoskeletal pain by increasing muscle strength and reducing pain sensitivity (25). Therefore, more research is needed to study different types of physical activity, such as vigorous-intensity activity, as potential mediators.

Our results indicate that working from home is associated with an increased risk of pain in the upper back and in the arm, neck, and/or shoulder. Correspondingly, three earlier studies reported an association between working from home and pain in these body parts (13, 14, 17). However, earlier studies also observed an association with pain in the lower back (13, 15–17), while this was unexpectedly not found in the current study. Still, in the studies that included multiple body regions, larger effect estimates were generally observed for pain in the upper back and pain in the arm, neck, and/or shoulder than for pain in the lower back (13, 14, 17). This was also observed in our earlier study (20). A possible explanation for this finding may be that working from home is associated with performing more screen work and being more sedentary than working on location (6–9, 11, 45), which is particularly related to pain in the upper part of the musculoskeletal system (39–41), although it may also negatively impact pain in the lower back. Prolonged sitting requires a continuous static load on the muscles of the upper musculoskeletal system (25). Therefore, in particular the upper body regions such as the higher back and arm, neck, and/or shoulders may be at risk of physical strain.

After 2 years of the COVID-19 pandemic, the work-from-home measure was lifted in The Netherlands and other countries. However, working from home has remained a common practice for many workers and the Dutch government currently still calls on employers to permanently encourage workers to work partly from home (46). This stresses the importance of permanently creating healthy work environments at home and encouraging workers to adopt healthy work-from-home practices. The results of the current study imply that supporting home workers in reducing sedentary time should be an important component of such healthy work-from-home practices, as it may contribute to preventing musculoskeletal pain. To this end, more research is needed to examine effective strategies and interventions to reduce home workers' sitting time. Promising interventions include height-adjustable desks (i.e., sit-stand desks) (47–49), digital tools (50, 51), and taking frequent short breaks (49). Policy makers and employers should develop and implement work-from-home policies that facilitate home workers in reducing sedentary time at their workplace and that aim to prevent musculoskeletal pain.

While sedentary behavior was found to be a relevant mediator in the association between working from home and pain in the upper musculoskeletal system, it only partly explained this association. This indicates that further work is needed to better understand other underlying mechanistic factors linking working from home and musculoskeletal pain. Such factors may for example be found in the home office environment and in psychosocial risk factors (12, 21, 52).

### Strengths and limitations

Strengths of the current study are its large sample size and its longitudinal design with many repeated measurements covering almost 2 years of the COVID-19 pandemic.

Since information on sedentary behavior and information on pain in the upper back/pain in the arm, neck, and/or shoulder was only collected from, respectively, round 6 and round 8 onwards, a substantial part of the total study population did not complete questions on these topics. Therefore, the research questions including these mediator and outcomes could only be studied in a subsample of the population. As a result, effect estimates describing the same association somewhat varied between subsamples of the study populations. Nevertheless, these effect estimates were generally of the same magnitude and in the same direction across these subsamples, supporting the robustness of our findings.

Working from home was found to be associated with increased sedentary behavior. It is likely that this increase in sedentary behavior associated with working from home is predominantly caused by an increase in screen work (14). However, the current study lacked information on screen work, screen time, or the working positions while performing screen work from home, so the potential pathway linking remote work to sedentary behavior *via* screen time could not be examined. Furthermore, screen work independent of sitting time could possibly negatively impact musculoskeletal system by causing overuse of muscles during computer use (41) Future work should contribute to elucidating these potential pathways.

In the current study, 500 bootstraps resamples were used to calculate the confidence intervals of the indirect effects. Preferably, this number should be at least 1,000 (53). However, due to the large sample size of the current study with up to over two hundred thousand observations, the number of possible bootstrap resamples was restricted by computational limits. As analyses with 50, 100, 200, and 500 bootstrap resamples all showed almost identical confidence intervals, we do not expect that further increasing the number of bootstrap resamples would have had a large impact on our results.

Due to the longitudinal design of the current study, it was possible to conduct a mediation analysis, because the temporal sequence of exposure and mediator on the one hand, and outcome one the other hand could be taken into account. An important assumption of mediation analysis is that there is no unmeasured confounding in the association between determinant and mediator, between mediator and outcome, and between determinant and outcome (54). To this end, a variety of covariates was included covering demographic factors, work-related factors, and general health as well as physical activity and sedentary behavior prior to the pandemic. Home and hybrid workers differed considerably from location workers with respect to characteristics such as education level and occupation (Table 1). Still, the same associations of working from home with physical activity and sedentary behavior and musculoskeletal pain were observed when stratifying the population based on these characteristics (11, 20). However, residual confounding cannot be ruled out. As this is the first study into the mediating role of physical activity and sedentary behavior in the association between working from home and musculoskeletal pain, more research is needed to replicate our findings.

## Conclusion

Compared to location workers, home and hybrid workers were more likely to have pain in the upper musculoskeletal system during the COVID-19 pandemic, which was partly mediated by increased sedentary behavior. Physical activity was not found to be a mediator in the association between working from home and musculoskeletal pain. Future home work policies should incorporate strategies aimed at reducing sedentary time in order to prevent musculoskeletal pain.

## Data availability statement

The data analyzed in this study is subject to the following licenses/restrictions: Obtained from a third party, i.e., Lifelines (https://www.lifelines.nl/researcher). Requests to access these datasets should be directed to Lifelines Research Office (research@lifelines.nl).

## Ethics statement

The studies involving human participants were reviewed and approved by the Medical Ethics Committee of the University Medical Center Groningen, The Netherlands (number 2007/152). The patients/participants provided their written informed consent to participate in this study.

## Lifelines Corona Research Initiative

H. Marike Boezen^1^^†^, Jochen O. Mierau^2, 3, 9^, H. Lude Franke^4^, Jackie Dekens^4, 6^, Patrick Deelen^4^, Pauline Lanting^4^, Judith M. Vonk^1^, Ilja Nolte^1^, Anil P.S. Ori^4, 5^, Annique Claringbould^4^, Floranne Boulogne^4^, Marjolein X.L. Dijkema^4^, Henry H. Wiersma^4^, Robert Warmerdam^4^, Soesma A. Jankipersadsing^4^, Irene van Blokland^4, 7^, Geertruida H. de Bock^1^, Judith GM Rosmalen^5, 8^, Cisca Wijmenga^4^

^1^Department of Epidemiology, University of Groningen, University Medical Center Groningen, Groningen, The Netherlands

^2^Department of Economics, Econometrics & Finance, Faculty of Economics and Business, University of Groningen, Groningen, The Netherlands

^3^Lifelines Cohort Study and Biobank, Groningen, The Netherlands

^4^Department of Genetics, University of Groningen, University Medical Center Groningen, Groningen, The Netherlands

^5^Department of Psychiatry, University of Groningen, University Medical Center Groningen, Groningen, The Netherlands

^6^Center of Development and Innovation, University of Groningen, University Medical Center Groningen, Groningen, The Netherlands

^7^Department of Cardiology, University of Groningen, University Medical Center Groningen, Groningen, The Netherlands

^8^Department of Internal Medicine, University of Groningen, University Medical Center Groningen, Groningen, The Netherlands

^9^Team Strategy & External Relations, University of Groningen, University Medical Center Groningen, The Netherlands

^†^Deceased

## Author contributions

BL analyzed the data and wrote the first draft of the manuscript. KP was the principal investigator of the study. All authors commented on previous versions of the manuscript and approved the final manuscript.

## Funding

This study was funded by the COVID-19 program of the Dutch Ministry of Health, Welfare and Sport that was conducted by the Dutch National Institute for Public Health and the Environment. The funding bodies had no role in the study design, the collection, analysis, interpretation of data, the writing of the manuscript, and the decision to submit the manuscript for publication.

## Conflict of interest

The authors declare that the research was conducted in the absence of any commercial or financial relationships that could be construed as a potential conflict of interest.

## Publisher's note

All claims expressed in this article are solely those of the authors and do not necessarily represent those of their affiliated organizations, or those of the publisher, the editors and the reviewers. Any product that may be evaluated in this article, or claim that may be made by its manufacturer, is not guaranteed or endorsed by the publisher.
