# Supplementary material for: The mediating role of physical activity and sedentary behavior in the association between working from home and musculoskeletal pain during the COVID-19 pandemic
Source: Front Public Health. 2022 Dec 2;10:1072030. doi: 10.3389/fpubh.2022.1072030 (PMC9757165; doi:10.3389/fpubh.2022.1072030)
Supplement: Supplementary file 1 [file Data_Sheet_1.pdf]

Supplementary Material

1 Supplementary Tables

Table S1. Overview of the used questions in the current study from the questionnaire rounds of the Lifelines COVID-19 cohort from March 2020 to January 2022.

Round	Date	Determinant: work situation question	Mediators: physical activity (PA) and sedentary behavior (SB) questions	Outcomes: Pain in lower back (LB), upper back (UB), arm, neck, and/or shoulder (ANS) questions
1	Mar to Apr 2020	Work situation	PA	-
2	Apr to May 2020	Work situation	PA	Pain in LB
3	Apr to May 2020	Work situation	PA	Pain in LB
4	Apr to May 2020	Work situation	PA	Pain in LB
5	Apr to May 2020	Work situation	PA	Pain in LB
6	Apr to May 2020	Work situation	PA + SB	Pain in LB
7	May 2020	Work situation	PA	Pain in LB
8	May to Jun 2020	Work situation	PA	Pain in LB + UB + ANS
9	Jun 2020	-	-	Pain in LB + UB + ANS
10	Jul 2020	-	-	-
11	Jul to Aug 2020	Work situation	PA + SB	-
12	Jul to Sep 2020	-	-	Pain in LB + UB + ANS
13	Sep 2020	-	-	-
14	Oct to Nov 2020	Work situation	PA + SB	-
15	Nov 2020	Work situation	PA + SB	Pain in LB + UB + ANS
15b	Nov to Dec 2020	-	-	Pain in LB + UB + ANS
16	Dec 2020	Work situation	PA + SB	-
16b	Dec 2020 to Jan 2021	-	-	Pain in LB + UB + ANS
17	Jan to Feb 2021	Work situation	PA + SB	-
18	Feb to Mar 2021	-	-	Pain in LB + UB + ANS
19	Mar to Apr 2021	Work situation	PA + SB	-
20	Apr to May 2021	-	-	Pain in LB + UB + ANS
21	May to Jun 2021	Work situation	PA + SB	-
22	Jul 2021	-	-	Pain in LB + UB + ANS
23	Oct to Nov 2021	Work situation	PA + SB	-
24	Dec 2021 to Jan 2022	-	-	Pain in LB + UB + ANS

Table S2. Characteristics of the study population stratified for location workers, home workers, and hybrid workers (who worked both on location and from home) during the COVID-19 pandemic from March 2020-January 2022 (n=28,586)

	**Location workers (n=13,489)**		**Home workers (n=5,971)**		**Hybrid workers (n=9,126)**	
	Mean/%	SD/n	Mean/%	SD/n	Mean/%	SD/n
Age (in years)	50.9	8.6	49.3*	9.1	49.1ɫ	9.3
Sex (% female)	61.3	8264	56.6*	3377	60.3	5503
Educational level (%)						
Low	20.8	2807	5.3*	317	4.9ɫ	445
Middle	55.7	7511	28.9*	1724	30.4ɫ	2778
High	23.5	3171	65.8*	3930	64.7ɫ	5903
Household composition (%)						
Living alone	7.8	1058	8.1	484	6.9ɫ	628
Living together with child(ren)	1.4	191	2.4*	141	1.9ɫ	172
Living together with adult(s)	53.8	7251	47.5*	2838	47.7ɫ	4354
Living together with child(ren) and adult(s)	35.8	4829	40.8*	2438	42.4ɫ	3866
Living together but unknown with whom	1.2	160	1.2	70	1.2	106
Born in the Netherlands (% yes)	98.2	13248	97.5*	5821	98.2	8964
Occupation (%)						
High-skilled white-collar	42.2	5693	73.2*	4373	72.4ɫ	6605
Low-skilled white-collar	34.2	4607	23.2*	1388	22.3ɫ	2039
High-skilled blue-collar	11.2	1515	2.1*	128	3.5ɫ	323
Low-skilled blue-collar	12.4	1674	1.4*	82	1.7ɫ	159
Occupational class (%)						
Educational occupations	2.7	358	8.3*	495	16.4ɫ	1500
Creative and linguistic occupations	0.8	102	3.1*	184	2.4ɫ	216
Commercial occupations	9.0	1220	8.0*	476	6.7ɫ	615
Business economics and administrative occupations	15.7	2114	37.9*	2262	27.4ɫ	2505
Managers	3.9	530	5.7*	343	6.7ɫ	616
Public administration, security, and legal occupations	2.4	330	6.6*	392	4.5ɫ	411
Technical occupations	15.8	2126	6.9*	410	8.7ɫ	793
ICT occupations	1.0	132	11.5*	687	5.0ɫ	455
Agricultural occupations	1.7	236	1.1*	66	1.6	144
Care and welfare occupations	33.2	4482	8.1*	482	17.2ɫ	1567
Service occupations	8.3	1115	2.4*	141	2.6ɫ	237
Transport and logistics occupations	5.2	708	0.5*	32	0.7ɫ	63
Employment contract (%)						
Permanent contract	79.1	10669	75.0*	4479	70.2ɫ	6404
Permanent and non-permanent contract	8.9	1202	6.3*	377	14.2ɫ	1293
Non-permanent contract	12.0	1618	18.7*	1115	15.7ɫ	1429
General health (% excellent/(very) good)	96.5	13019	95.3*	5693	95.9ɫ	8748

≥150 minutes of physical activity per week before COVID-19 pandemic (% yes)	41.9	5651	44.6*	2662	42.1	3838
Sitting >9 hours per work day before COVID-19 pandemic (% yes)[1]	12.1	913	43.3*	1274	30.4*	1902

* Statistically significant difference ($p<0.05$) between home workers and location workers tested with independent-samples t-test and chi-square test.

ł Statistically significant difference ($p<0.05$) between hybrid workers and location workers tested with independent-samples t-test and chi-square test.

1 Among 7573 locations workers, 2943 home workers, and 6264 hybrid workers.

2 Supplementary Figures

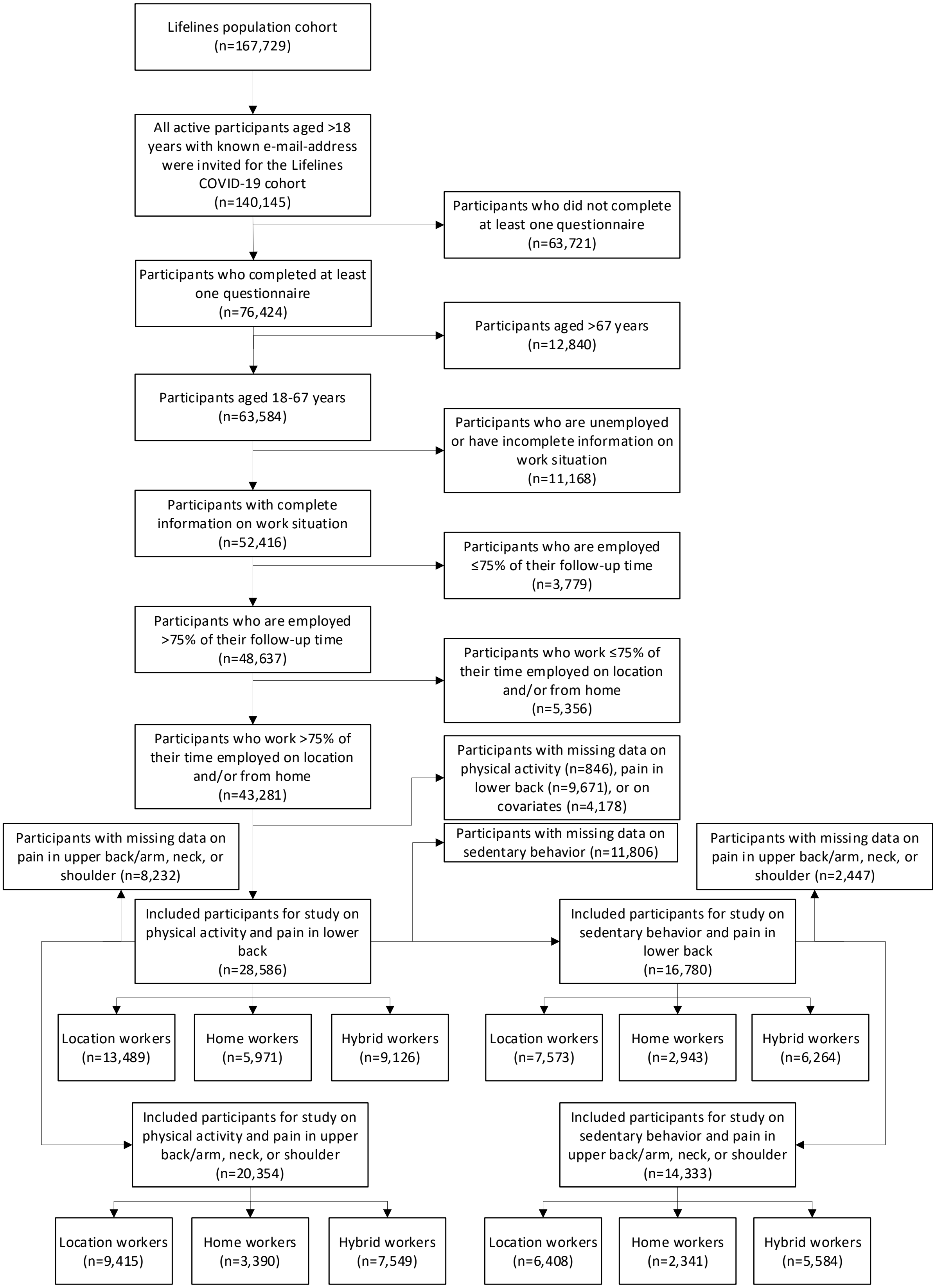

Figure S1. Flowchart of study participants

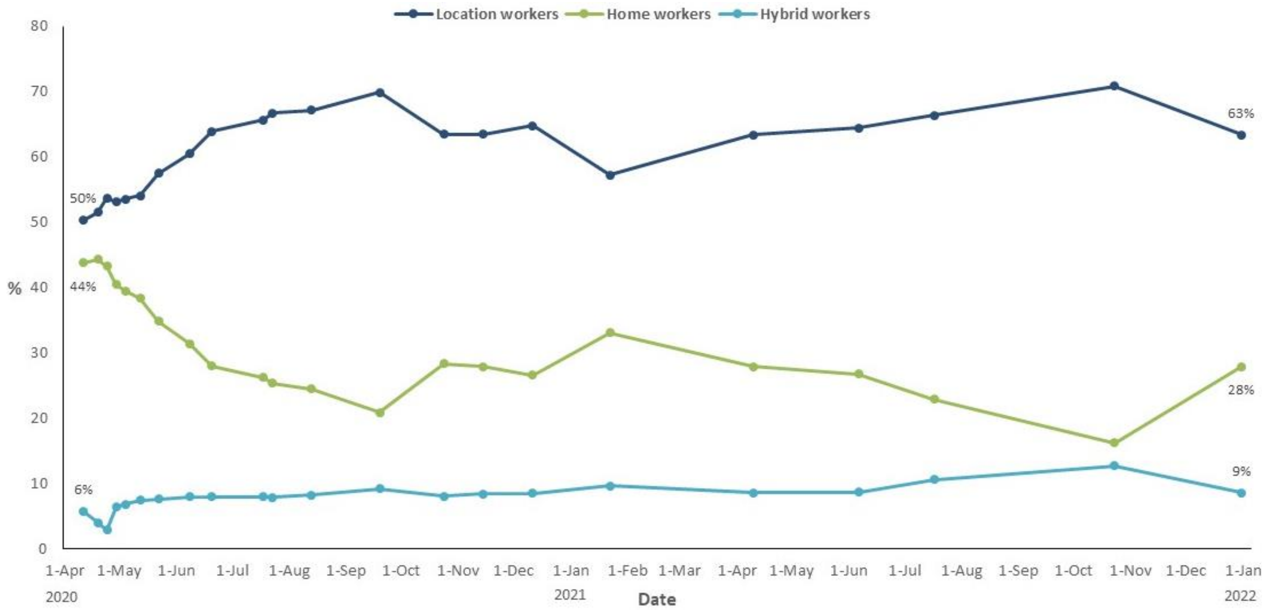

Figure S2. Percentages of location workers, home workers, and hybrid workers at 24 questionnaire rounds during the study period (March 2020–January 2022) among 28,586 workers. Every datapoint represents the median date of the particular questionnaire round.
